# Cost comparison between uterine-sparing fibroid treatments one year following treatment

**DOI:** 10.1186/2050-5736-2-7

**Published:** 2014-03-31

**Authors:** Bijan J Borah, Ginger S Carls, Brian J Moore, Teresa B Gibson, James P Moriarty, Elizabeth A Stewart

**Affiliations:** 1 Division of Health Care Policy and Research & College of Medicine, Mayo Clinic, 200 First Street SW, Rochester, MN 55905, USA; 2 Truven Health Analytics, 777 E. Eisenhower Parkway, Ann Arbor, MI 48108, USA; 3 Division of Health Care Policy and Research, Mayo Clinic, Rochester, Minnesota, USA; 4 Division of Reproductive Endocrinology, Department of Obstetrics and Gynecology and Department of Surgery, Mayo Clinic and Mayo Medical School, Rochester, Minnesota, USA

**Keywords:** Healthcare costs, Myomectomy, Uterine artery embolization (UAE), Uterine fibroids, Magnetic resonance-guided focused ultrasound (MRgFUS)

## Abstract

**Background:**

To compare one-year all-cause and uterine fibroid (UF)-related direct costs in patients treated with one of the following three uterine-sparing procedures: magnetic resonance-guided focused ultrasound (MRgFUS), uterine artery embolization (UAE) and myomectomy.

**Methods:**

This retrospective observational cohort study used healthcare claims for several million individuals with healthcare coverage from employers in the *MarketScan* Database for the period 2003–2010. UF patients aged 25–54 on their first UF procedure (index) date with 366-day baseline experience, 366-day follow-up period, continuous health plan enrollment during baseline and follow-up, and absence of any baseline UF procedures were included in the final sample. Cost outcomes were measured by allowed charges (sum of insurer-paid and patient-paid amounts). UF-related cost was defined as difference in mean cost between study cohorts and propensity-score-matched control cohorts without UF. Multivariate adjustment of cost outcomes was conducted using generalized linear models.

**Results:**

The study sample comprised 14,426 patients (MRgFUS = 14; UAE = 4,092; myomectomy = 10,320) with a higher percent of older patients in MRgFUS cohort (71% vs. 50% vs. 12% in age-group 45–54, *P* < 0.001). Adjusted all-cause mean cost was lowest for MRgFUS ($19,763; 95% CI: $10,425-$38,694) followed by myomectomy ($20,407; 95% CI: $19,483-$21,381) and UAE ($25,019; 95% CI: $23,738-$26,376) but without statistical significance. Adjusted UF-related costs were also not significantly different between the three procedures.

**Conclusions:**

Adjusted all-cause and UF-related costs at one year were not significantly different between patients undergoing MRgFUS, myomectomy and UAE.

## Introduction

Uterine fibroids (UF) are benign clonal tumors affecting more than one-fifth of all women of reproductive age in the U.S [1,2]. Although benign, UF often occur with severe symptoms including pelvic pain, prolonged periods with heavy bleeding, bladder pressure, and adverse reproductive outcomes [3-5].

UF-related morbidity has significant economic implications with direct costs ranging from $5,395 to $9,610 (in adjusted 2010 U.S. Dollars) [1,6,7]. In addition, indirect costs of UF (e.g., missed work) are significantly higher at $12,930 versus $8,893 for controls (also adjusted to 2010 values) [6,7]. The overall economic burden of UF in the U.S. has been estimated to be between $5.9-34.4 billion (in 2010 U.S. Dollars) annually.

Among the available alternatives to hysterectomy for UF are endoscopic and abdominal myomectomy, uterine artery embolization (UAE) and magnetic resonance-guided focused ultrasound (MRgFUS). Several studies have assessed the clinical and patient-related outcomes between these three procedures [8-14]. However, as documented in a recent Agency for Healthcare Research and Quality publication, critical gaps exist with regard to relative costs of available treatment options for UF including MRgFUS, UAE and myomectomy [3]. Comparison of costs of MRgFUS, the newest of these options, is complicated by the fact that major U.S. commercial insurance carriers do not generally include MRgFUS as a covered UF treatment option which in turn results in dearth of cost data for MRgFUS [15-17]. The use of a large multi-payer database as used in this study may circumvent the issue of lack of cost data for MRgFUS, and provide the best possible evidence on cost comparison between the three competing UF treatment procedures. As such, this study aims to compare both (i) all-cause direct healthcare costs and (ii) UF-related direct healthcare costs between MRgFUS, UAE and myomectomy cohorts one year following treatment. This study will use reimbursed amounts by commercial payers as healthcare costs, and thus provide real-world evidence of relative costs between the three uterine-sparing procedures for UF treatment.

## Methods

### Study design & data source

The study was a retrospective cohort analysis of healthcare costs using claims from *MarketScan Commercial Claims and Encounters* database, providing enrollment data and paid medical claims for patients insured primarily through large self-insured employers. MarketScan database contains inpatient and outpatient healthcare utilization, and outpatient prescription drug experience of several million employees and their dependents (annually), covered under a variety of fee-for-service and capitated health plans, including exclusive provider organizations, preferred provider organizations (PPOs), point of service (POS) plans, indemnity plans, and health maintenance organizations (HMOs). MarketScan database also provides detailed cost and outcomes data associated with healthcare service and drug utilization described above. This is facilitated by linking medical claims for each patient to person-level enrollment data and to outpatient prescription drug claims through the use of unique enrollee identifiers.

### Study population and cohorts

**Figure 1:**
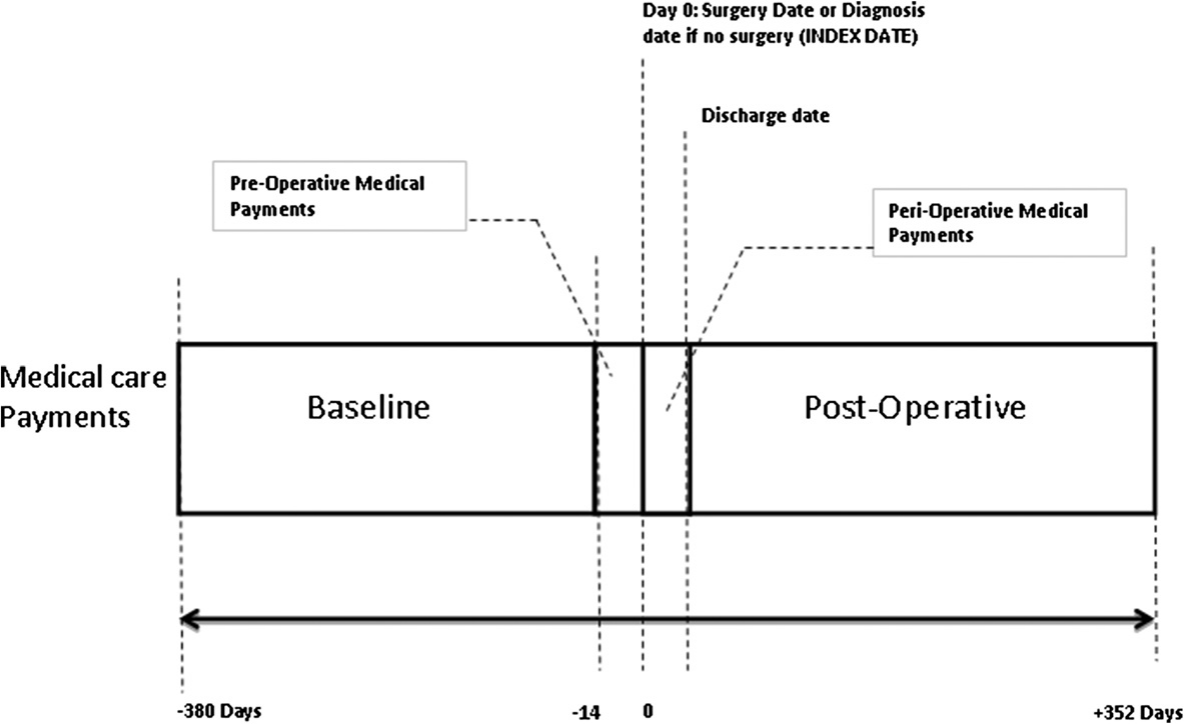
**Study period for women with surgery/procedure for UF treatments.** This figure delineates the various study periods, including the baseline and the follow-up operative period.

UF patients aged 25–54 years as of their first UF procedure date (*index date*) between 2003 and 2010 were used in the study. Since MRgFUS was approved by FDA in 2004 and UAE and myomectomy were approved much earlier than that, we considered all patients who had a UF procedure between 2004 and 2009. Since we required each patient to have 1-year baseline and 1-year follow-up, the possible date range for included claims was 2003 through 2010. Similar to Carls et al [18]. and as shown in Figure 1, healthcare costs were assessed for a 1-year baseline period, and for each of the following segments of the 1-year operative period: *pre-operative* (14 days prior to the index date), *peri-operative* (from the index date until discharge) and *post-operative* (from the discharge date until 1 year after the start of the pre-operative period). The three study cohorts were defined based on the index UF procedure: MRgFUS, UAE and myomectomy (see Table 1 for relevant CPT codes).

**Table 1:** CPT codes for identifying index cohorts

**Treatment category**		**CPT-4 codes**
**The 3 study cohorts**		
Magnetic Resonance Focused Ultrasound (MRgFUS)		0071T, 0072T
Uterine Artery Embolization (UAE)^*^		36247, 37204, 52250, 37210 and require UF dx on claim for each of the codes
Myomectomy		58140, 58146
Abdominal		58140, 58146
Vaginal		58145
Laparoscopic or robotic		58545,58546, 58551
**Procedure codes used in exclusion criteria**		
Endometrial ablation		56356, 58353, 58563
Excision/destruction of lesion of uterus		58561
Hysterectomy		
Total abdominal		58150, 58152, 58200,58953, 58954, 58956
Vaginal		58260,58262, 58263, 58267, 58270, 58275, 58280, 58285, 58290-58294
Laparoscopic or robotic		58550, 58552, 58553, 58554, 58541, 58542, 58543, 58544, 58570, 58571, 58572, 58573, 58578^*^
Subtotal		58180
Radical^*^		58210, 58548 and require UF diagnosis on claim for each of the codes

Notes:

^*^Require a UF diagnosis on the claim with the CPT4 code for all of the Radical Hysterectomy and UAE CPT4 codes, plus CPT4 code 58578. UF diagnosis = ICD-9-CM dx codes 218.xx, 219.xx or 654.1x.

In addition, patients included in the study must have: a UF diagnosis during the baseline period (see Table 2 for ICD-9 diagnosis codes); continuous enrollment in the health plan during the baseline and follow-up periods; and absence of any UF treatment procedure during baseline (see Table 1 for relevant codes).

**Table 2:** Diagnosis codes for uterine fibroids and related complications/comorbidities

**Diagnoses**	**ICD-9-CM diagnosis codes**
Uterine fibroids	218.xx, 219.xx, 654.1x
Menstruation disorders	626.xx
Pelvic pain	625.xx
Anemias	280.xx, 285.xx
Inflammatory diseases	614.xx, 616.xx
Noninflammatory diseases	620.xx, 622.xx
Endometriosis	617.xx
Pregnancy	V22.x, V23.x, V27.x, 640.00-677
Urinary problems	788.41, 788.20-788.29, 591.xx, 593.5x
Constipation or gas	564.00, 564.09, 787.3
Disorders of the uterus not elsewhere classified	621.xx
Genital prolapse	618.xx
Benign neoplasm of the uterus/ovary	220.xx

### Patient characteristics

Patient characteristics included index age reported in three age categories (25–34, 35–44 and 45–54), geographic region of residence of the primary enrollee (e.g. employee), whether the patient was the primary enrollee or the spouse or other dependent, year of the index date, and type of health plan. Baseline health status was measured using the Charlson Comorbidity Index (CCI) [19], a count of the number of psychiatric diagnostic groupings [20], and the presence of certain diagnoses during the baseline period (menstrual disorders, pelvic pain, anemia, inflammatory disease, non-inflammatory disease, endometriosis, pregnancy, urinary problems, constipation or gas, other disorders of the uterus, genital prolapse, benign neoplasm of the uterus, infertility and breast cancer). Furthermore, indicators for any inpatient and emergency room (ER) visits during baseline were extracted to proxy for potentially higher baseline risk in those patients and hence the potential for higher costs in the follow-up period not necessarily related to UF. Pharmacotherapy use (non-steroidal anti-inflammatories or NSAIDs, and hormone therapy) during baseline was also captured. The choice of treatment and consequent costs may be confounded by patients’ socioeconomic characteristics including income, education and race. Because these variables are not available in insurance claims, data on median income, percent of black residents and percent of residents with college education in the primary enrollee’s 5-digit ZIP code of residence on index date were used as proxies based on 2010 census data.

### Study outcomes

The primary study outcome was all-cause direct healthcare cost in 2010 U.S. dollars (inflation adjusted using the GDP Implicit Price Deflator), which included both insurer-paid amounts and patient-paid amounts (including copayments and deductibles) for all the claims generated through the 1-year follow-up period as has been done in previous studies [1,18]. All-cause cost was also compared between the three study cohorts for each of the pre-operative, peri-operative and 1-year post-operative periods separately.

Another outcome assessed was UF-related direct cost defined as the difference in mean costs between a study cohort and its control cohort that included similar patients from MarketScan database but without a UF diagnosis. At a minimum, patients in the control cohorts must satisfy following criteria: at least 12 months enrollment before and after the index date (randomly assigned date to match the distribution of index dates among patients with UF), be of age 25–54 on their index date, and did not have any UF diagnoses in their claims history. One-to-one propensity score matching [21,22] was used to construct the comparison cohorts for myomectomy and UAE using the all the variables described in the patient characteristics section. However, there were not sufficient degrees of freedom to use this approach for the 14 MRgFUS patients. Instead, each MRgFUS patient was exactly matched to five comparison patients using a subset of patient characteristics: age (±5 years), Census region, health plan type, index year, income, education, race, an indicator for baseline CCI > 0 (described below) and an indicator for baseline inpatient admission.

### Analytic strategy

Descriptive statistics for baseline characteristics, including mean and standard deviation (SD) for continuous covariates, and frequency and percent for categorical variables, are provided for each of the three study cohorts.

Selection of women into each treatment cohort is likely to be the main source of bias. MRgFUS is not accessible to many patients since this treatment is considered investigational by most U.S. insurers. We address potential selection bias in two ways. First, we describe the characteristics of women in each sample, including comorbidities and cost prior to surgery, since comorbidities may influence treatment choices. Second, we adjust costs to control for observed differences between patients in each treatment group through multivariate regression.

### Multivariate adjustment of cost outcomes

Costs were regression-adjusted to control for baseline characteristics of women in each treatment group. This was implemented using generalized linear modeling (GLM) framework with a gamma distribution and logarithmic link [23,24]. Separate GLM models were constructed for costs in each time period, and included indicators for each treatment cohort and all patient characteristics described above as independent variables. Predicted costs from these regressions are reported for each of the study groups.

Mayo Clinic Institutional Review Board considered this study as exempt from full review given that study used already existing deidentified data. The analytic dataset was created using SAS (version 9.2), while the analyses were conducted in Stata (version 11).

## Results

**Figure 2:**
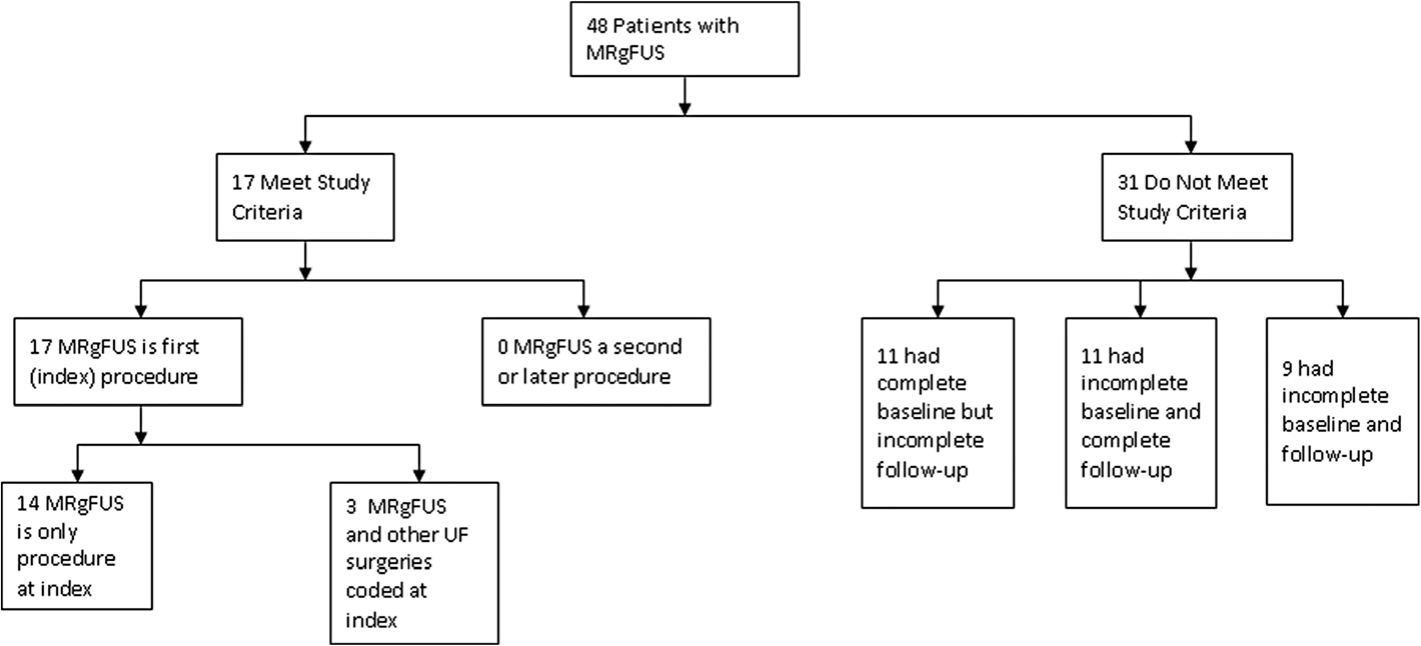
**Patient Selection in the MRgFUS Cohort.** This figure shows attrition of patients in the MRgFUS cohort due to each inclusion or exclusion criterion.

For the primary analysis, 14,426 patients with uterine-sparing procedures (MRgFUS = 14; UAE = 4,092 and myomectomy = 10,320) met study criteria. For the secondary analysis of fibroid-related costs, a matched group of 14,482 patients without UF were selected (70 MRgFUS, 4,092 UAE, and 10,320 myomectomy). Given that only 14 of the 48 MRgFUS patients found in the *MarketScan* database met all our criteria for length of enrollment, we provided a flowchart in Figure 2 to document the attrition of patients due to each inclusion/exclusion criterion. In addition, Table 3 describes the characteristics of the 48 MRgFUS patients found in the database. The 14 MRgFUS patients who met study criteria were similar to the original sample of 48 MRgFUS patients in terms of the baseline characteristics (see Table 4 and Table 3).

**Table 3:** Description of MRgFUS patients (N = 48) and procedures in data (N = 54)

**MRgFUS patients (N = 48)**	**N**	**%**
**Age of patient**		
25-34	2	4.2%
35-44	20	41.7%
45-54	25	52.1%
55-64	1	2.1%
**Plan type**		
Fee for service	2	4.2%
Preferred Provider Organization (PPO)	27	56.3%
Point of Service (POS)	4	8.3%
Health Maintance Organizations (HMO)	9	18.8%
Capitated POS	1	2.1%
Consumer Driven Health Plan (CDHP)	4	8.3%
Unknown	1	2.1%
**Sociodemographics of ZIP Code of residence**		
Median household income		
Quintile 1 ($0,$28,280)	2	4.2%
Quintile 2 ($28,281, $33,680)	1	2.1%
Quintile 3 ($33,681, $39,204)	6	12.5%
Quintile 4 ($39,205, $48,749)	14	29.2%
Quintile 5 (≥$48,750)	25	52.1%
% over 25 with college degree		
Quintile 1 (0-7%)	1	2.1%
Quintile 2 (8-11%)	0	0.0%
Quintile 3 (12-15%)	10	20.8%
Quintile 4 (16-24%)	11	22.9%
Quintile 5 (≥25%)	26	54.2%
% Black		
Quintile 1 (0%)	0	0.0%
Quintile 2 (0.1-0.3%)	2	4.2%
Quintile 3 (0.4-1.3%)	6	12.5%
Quintile 4 (1.4-8.5%)	27	56.3%
Quintile 5 (≥8.6%)	13	27.1%
**Employment details**		
Employee (not spouse or dependent)	35	72.9%
Active fulltime	24	50.0%
Salaried	13	27.1%
**MRgFUS procedures (N = 54)**		
**Cost of first procedure (N = 48)**		**$**
Employer		$6,425
Out-of-pocket		$902
Mean		$7,327
Std. Dev.		$6,521
**Year of procedure**		
2004	0	0.0%
2005	3	5.6%
2006	7	13.0%
2007	11	20.4%
2008	13	24.1%
2009	12	22.2%
2010	8	14.8%
**Surgical setting**	**N**	**%**
Inpatient	1	1.9%
Outpatient	53	98.1%
**Inclusion or exclusion in study sample**	**N**	**%**
Met study inclusion criteria	14	25.9%
Excluded from study sample		
Had other surgeries coded at index (multiple procedures)	3	5.6%
Incomplete baseline and/or follow-up	31	57.4%
Not the index procedure (Patient had a prior surgical treatment for UF)	6	11.1%

**Table 4:** Characteristics of patients treated with MRgFUS, UAE and Myomectomy

**Patient characteristics**	**MRgFUS**	**UAE**	**Myomectomy**	**P-Value**^**a**^
	**(N = 14)**	**(N = 4,092)**	**(N = 10,320)**	
**Age**				
25-34	0.0%	4.0%	33.1%	
35-44	28.6%	46.4%	55.3%	
45-54	71.4%	49.7%	11.6%	0.000
**Region**				
Northeast	NR	13.8%	14.2%	
Midwest	NR	18.8%	15.0%	
South	NR	52.4%	54.5%	
West	NR	14.2%	15.7%	
Unknown	NR	0.7%	0.6%	0.000
**Employment details**				
Employee (not spouse or dependent)	64.3%	71.7%	77.5%	0.000
**Plan type**				
Fee for service	7.1%	3.9%	3.0%	
HMO	28.6%	29.3%	24.4%	
Point of Service (POS)	7.1%	13.0%	15.0%	
Preferred Provider Organization	57.1%	47.9%	51.7%	
Capitated POS	0.0%	2.4%	2.0%	
Consumer Driven Health Plan (CDHP)	0.0%	2.1%	2.6%	0.000
**Year of index date**				
2004	0.0%	7.4%	10.8%	
2005	7.1%	13.8%	13.4%	
2006	14.3%	15.2%	14.4%	
2007	28.6%	16.5%	17.1%	
2008	21.4%	19.0%	19.5%	
2009	21.4%	27.4%	24.2%	0.000
**Sociodemographics of ZIP Code of residence**				
Median household income				
Quintile 1 ($0,$28,280)	0.0%	8.3%	9.2%	
Quintile 2 ($28,281, $33,680)	7.1%	8.3%	9.5%	
Quintile 3 ($33,681, $39,204)	14.3%	16.4%	16.5%	
Quintile 4 ($39,205, $48,749)	35.7%	19.2%	21.1%	
Quintile 5 (≥$48,750)	42.9%	47.8%	43.7%	0.001
% over 25 with college degree				
Quintile 1 (0-7%)	0.0%	0.2%	0.3%	
Quintile 2 (8-11%)	0.0%	2.3%	2.6%	
Quintile 3 (12-15%)	14.3%	13.5%	13.2%	
Quintile 4 (16-24%)	71.4%	29.7%	31.0%	
Quintile 5 (≥25%)	14.3%	54.2%	52.9%	0.333
% Black				
Quintile 1 (0%)	0.0%	3.7%	3.5%	
Quintile 2 (0.1-0.3%)	0.0%	7.3%	7.8%	
Quintile 3 (0.4-1.3%)	28.6%	15.5%	15.6%	
Quintile 4 (1.4-8.5%)	14.3%	22.2%	23.6%	
Quintile 5 (≥8.6%)	57.1%	51.2%	49.5%	0.050
**Surgical setting**				
Inpatient	0.0%	18.3%	74.6%	
Outpatient	100.0%	81.7%	25.4%	0.000
**Use of pharmacotherapy during baseline**				
NSAIDs (non-steroidal anti-inflammatories)	35.7%	27.9%	24.9%	0.001
Hormone therapy	42.9%	31.1%	40.5%	0.000
**Healthcare use during baseline**				
Any inpatient visits	14.3%	5.7%	7.0%	0.010
Any ER visits	50.0%	20.8%	24.4%	0.000
**Baseline health status**				
Avg. Charlson Cormorbidity Index	0.14	0.23	0.17	
Charlson Cormorbidity Index >0	14.3%	16.0%	12.8%	0.000
Avg. Number of Psychiatric Diagnostic Groupings	2.57	2.06	2.81	
Number of Psychiatric Diagnostic Groupings >1	50.0%	58.5%	76.4%	0.000
Menstrual disorders	64.3%	56.3%	45.9%	0.000
Pelvic pain	42.9%	24.0%	31.3%	0.000
Anemias	7.1%	28.4%	17.9%	0.000
Inflammatory disease	28.6%	14.2%	18.4%	0.000
Noninflammatory disease	7.1%	14.0%	17.6%	0.000
Endometriosis	0.0%	2.2%	5.2%	0.000
Pregnancy	7.1%	2.9%	15.2%	0.000
Urinary problems	0.0%	3.4%	3.7%	0.508
Constipation or gas	7.1%	2.8%	2.9%	0.605
Other disorders of the uterus	14.3%	19.4%	13.3%	0.000
Genital prolapse	0.0%	0.5%	0.6%	0.745
Benign neoplasm of the uterus	0.0%	0.1%	0.5%	0.003
Infertility	0.0%	1.0%	14.8%	0.000
Breast cancer	0.0%	0.6%	0.5%	0.729
a. P-value from chi-square test of significance of difference between the groups				
NR = not reportable due to small sample size				

Table 4 compares baseline characteristics of the three study cohorts. Patients in the three cohorts differed in their age distribution, with higher percentage of MRgFUS patients (71%) in the age group 45–54 compared to 50% and 12% in UAE and myomectomy cohorts, respectively. There was no patient in the 25–34 age group that received MRgFUS. Although geographic distribution of MRgFUS patients could not be reported in order to protect privacy, the corresponding p-value suggests that the three study cohorts were different with regard to their geographic distribution. The distribution of employee status (whether primary beneficiary or dependent) between the study cohorts also differed with 64% of MRgFUS patients were employees as opposed to 72% and 78% of UAE and myomectomy patients, respectively. The three study cohorts also differed with respect to the distribution of health plan types and the index year.

The three cohorts were different with regard to the their ZIP code-level income distribution, with a higher percentage of women in the MRgFUS cohort (79%) coming from areas with income greater than $39,204 compared to 67% and 65% of the patients in the UAE and myomectomy cohorts, respectively. The distribution of proportion of black residents in ZIP code-level regions from which the study patients came from differed between the three study cohorts, as did the surgical setting (outpatient versus inpatient) for the patients in the three cohorts.

Baseline health status, as captured by whether CCI > 0, differed significantly between cohorts (MRgFUS = 14%, UAE = 16%, Myomectomy = 13%, p-value < 0.001). However, other data appeared to suggest that women in the MRgFUS cohort may have more active or severe UF disease. For example, the three study cohorts were different with respect to their baseline NSAID use (MRgFUS = 36%, UAE = 28%, Myomectomy = 25%, p-value = 0.01), hormone therapy use (MRgFUS = 43%, UAE = 31%, Myomectomy = 41%, p-value < 0.001), baseline inpatient (IP) visits (MRgFUS = 14%, UAE = 6%, Myomectomy = 7%, p-value = 0.01), and baseline emergency room (ER) use (MRgFUS = 50%, UAE = 21%, Myomectomy = 24%, p-value < 0.001). Compared to women in UAE and myomectomy cohorts, women undergoing MRgFUS were also more likely to have menstrual disorders and pelvic pain but less anemia (all p < 0.01). Table 4 displays other baseline health conditions that exhibited significant differences between the three cohorts.

### Treatment costs

Table 5 reports average costs (all-cause) during the study period for each treatment group, both unadjusted and regression adjusted. One-year average healthcare costs for MRgFUS, UAE and myomectomy cohorts were $17,719, $18,638 and $16,879, respectively, and were not statistically different. While pre-operative costs were similar between the three cohorts, peri-operative costs for MRgFUS ($6,301, 95% CI: $2,572, $10,030) was significantly lower than for UAE ($11,444, 95% CI: $11,038, $11,849) and myomectomy ($10,555, 95% CI: 10,389, $10,721). Average post-operative costs for MRgFUS ($10,854, 95% CI: $3,573, $18,136) was higher than that for UAE ($6,408, 95% CI: $5,924, $6,891) and for myomectomy ($5,766, 95% CI: $5,550, $5,982) cohorts. The higher post-operative costs for the MRgFUS cohort was primarily driven by more outpatient visits (on average, 6.21 for MRgFUS vs. 5.7 for UAE and 5.5 for myomectomy) resulting in higher outpatient services cost for this cohort (on average, $5,784 for MRgFUS vs. $3,800 for UAE and $3,355 for myomectomy). Additionally, 2 of the 14 MRgFUS patients underwent a second UF procedure during the 1-year follow-up resulting in inpatient costs of $19,307 and $32,838, respectively. These costs for just 2 patients skewed the mean inpatient cost for the entire MRgFUS cohort by more than double the mean cost of the other two cohorts (on average, $3,725 for MRgFUS vs. $1,490 for UAE and $1,206 for myomectomy).

**Table 5:** Mean Costs for each treatment group for each time period (95% confidence intervals)

	**MRgFUS**	**UAE**	**Myomectomy**
	**(n = 14)**	**(n = 4,092)**	**(n = 10,320)**
**Unadjusted costs**			
Baseline	$11,562	$6,377	$6,241
	($3,039, $20,085)	($6,107, $6,648)	($6,016, $6,465)
Operative year (pre-, peri- and post-operative costs)	$17,719	$18,638	$16,879
	($10,068, $25,370)	($17,943, $19,332)	($16,592, $17,167)
Pre-operative	$564	$786	$558
	($18, $1,110)	($707, $866)	($521, $595)
Peri-operative	$6,301	$11,444	$10,555
	($2,572, $10,030)	($11,038, $11,849)	($10,389, $10,721)
Post-operative	$10,854	$6,408	$5,766
	($3,573, $18,136)	($5,924, $6,891)	($5,550, $5,982)
Change in costs from baseline to operative year	$6,157	$12,260	$10,638
	(−$5,294, $17,608)	($11,565, $12,955)	($10,351, $10,925)
Regression adjusted costs^*^			
Baseline	$9,901	$8,048	$6,991
	($5,562, $17,628)	($7,673, $8,439)	($6,691, $7,302)
Operative year (pre-, peri- and post-operative costs)	$19,763	$25,019	$20,407
	($10,425, $38,694)	($23,738, $26,376)	($19,483, $21,381)
Pre-operative	$556	$1,040	$629
	($19, $6,087)	($779, $1,388)	($485, $815)
Peri-operative	$7,626	$16,261	$13,938
	($187, $10,829)	($11,799, $22,400)	($10,330, $18,808)
Post-operative	$11,581	$7,718	$5,840
	($4,360, $30,765)	($7,122, $8,372)	($5,427, $6,279)
Change in costs from baseline to operative year	$9,862	$16,971	$13,416
	(−$435, $20,159)	($15,636, $18,306)	($12,445, $14,387)

^*^Adjusted costs employed regression models (generalized linear model with gamma distribution and logarithmic link) to control for baseline differences between women receiving each treatment. The regression included the following control variables, all measured on the index date or during the 1 year baseline period: age, region, type of health plan, year of procedure, whether the patient was an employee or dependent, whether or not the employee was active full-time, whether the employee was salaried, demographics characteristics of the ZIP Code where the patient lived, indicators for any outpatient prescription drug fills of hormone therapies and NSAIDs, the Deyo Charlson Comorbidity Index, number of psychiatric diagnostic groupings, indicators for comorbidities, and indicators for any hospital stays and any emergency department visits during the baseline year. Demographics of the ZIP code included quintile of zip code median family income, quintile of percent black and quintile of percent with a college degree.

Costs increased from baseline to the operative year the least for the MRgFUS group ($6,157), compared to the other treatments ($12,260 for UAE and $10,638 for myomectomy) although this was not a statistically significant difference.

Regression adjustment did not significantly alter the conclusions from the unadjusted analyses described above. Average adjusted operative year all-cause costs for MRgFUS, UAE and myomectomy were $19,763, $25,019 and $20,407, respectively and were not statistically different. Peri-operative costs were significantly lower for MRgFUS ($7,626) compared to UAE ($16,261) cohort; adjusted peri-operative cost for MRgFUS cohort was also lower than that for myomectomy ($13,938), but without statistical significance. The post-operative average cost was higher for MRgFUS ($11,581) than UAE ($7,718) and myomectomy ($5,840), although this difference was not statistically significant.

Table 6 presents average costs (unadjusted and regression adjusted) for each treatment group and their matched comparison during the operative year and shows fibroid-related costs as the difference between costs of the treated group and their matched comparison group. Unadjusted uterine fibroid related costs were highest for the MRgFUS group ($13,653) compared to the UAE ($10,839) and myomectomy ($7,906), although the differences between MRgFUS and the other two groups were not statistically significant. After regression adjustment, fibroid-related costs for MRgFUS ($14,614) were in between fibroid-related costs for UAE ($16,307) and myomectomy ($11,895) although the 95% confidence intervals for MRgFUS overlapped with those for the other two procedures, implying non-significant differences between the costs of the study procedures.

**Table 6:** Mean annual costs for treatment groups and comparison group, showing calculation of fibroid-related costs (95% confidence intervals)

	**MRgFUS**		**UAE**		**Myomectomy**	
	**Treated**	**Matched comparison**	**Treated**	**Matched comparison**	**Treated**	**Matched comparison**
	**(n = 14)**	**(n = 70)**	**(n = 4,092)**	**(n = 4,092)**	**(n = 10,320)**	**(n = 10,320)**
**Unadjusted costs**						
Operative costs (pre-, peri- and post-operative costs for treated, year after index date in comparison group)	$17,719	$4,066	$18,638	$7,798	$16,879	$8,973
	($10,068, $25,370)	($2,376, $5,755)	($17,943, $19,332)	($7,268, $8,328)	($16,592, $17,167)	($8,664, $9,283)
Uterine fibroid related costs in year after surgery (operative costs treated minus comparison group)	$13,653		$10,839		$7,906	
	($4,944, $22,362)		($10,358, $11,320)		($7,574, $8,328)	
Regression adjusted costs^*^						
Operative costs (pre-, peri- and post-operative costs for treated, year after index date in comparison group)	$19,763	$5,149	$25,019	$8,712	$20,407	$8,512
	($10,425, $38,694)	($3,883, $6,892)	($23,738, $26,376)	($8,290, $9,154)	($19,483, $21,381)	($8,092, $8,952)
Uterine fibroid related costs in year after surgery (operative costs treated minus comparison group)	$14,614		$16,307		$11,895	
	($7,469, $21,759)		($12,949, $19,665)		($7,996, $15,794)	

^*^Adjusted costs employed regression models (generalized linear model with gamma distribution and logarithmic link) to control for baseline differences between women receiving each treatment. The regression included the following control variables, all measured on the index date or during the 1 year baseline period: age, region, type of health plan, year of procedure, whether the patient was an employee or dependent, whether or not the employee was active full-time, whether the employee was salaried, demographics characteristics of the ZIP Code where the patient lived, indicators for any outpatient prescription drug fills of hormone therapies and NSAIDs, the Deyo Charlson Comorbidity Index, number of psychiatric diagnostic groupings, indicators for comorbidities, and indicators for any hospital stays and any emergency department visits during the baseline year. Demographics of the ZIP code included quintile of zip code median family income, quintile of percent black and quintile of percent with a college degree.

## Discussion

We found that one-year all-cause costs for MRgFUS ($19,763), myomectomy ($20,407) and UAE ($25,019) were not statistically different. Concerns have been raised about the costs of an image-guided procedure such as MRgFUS due to the costs of advanced imaging. Based on the data from this study, this concern does not appear to be justified.

This study significantly extends the literature on the comparability of MRgFUS costs compared to other fibroid treatments. The prior U.S. study that assessed cost-effectiveness of MRgFUS compared to other treatment options utilized expert opinions and clinical trial data yet found MRgFUS second only to hysterectomy in terms of cost-effectiveness [25]. Another study assessed cost-effectiveness of MRgFUS from the perspective of U.K.’s National Health Services [26]. In this system, a treatment strategy starting with MRgFUS at age 39 and following them to age 56 appears to be more cost-effective over a wide variety of assumptions than current practice [26]. Using real-world insurance claims data, our study appears to confirm prior findings that UF treatment costs are similar to UAE and myomectomy.

The significantly higher post-operative costs for MRgFUS seen in this study was initially puzzling given that MRgFUS is an outpatient procedure. Increased costs for both outpatient office visits and inpatient costs contribute to these higher MRgFUS costs. Given that MRgFUS is a relatively novel procedure, especially during the years of this study, it is possible that patients in this cohort were seen more frequently by their providers to monitor UF symptom relief. In addition, the small number of patients in the MRgFUS cohort magnifies the effect of two subjects going on to additional UF treatment on post-operative inpatient costs. Thus, the post-operative costs for MRgFUS cohort appear to be strongly skewed only by 2 of the MRgFUS patients, thereby rendering these costs less representative.

In addition to comparative cost estimates, the three study cohorts also exhibited some other notable trends. For example, a higher proportion of older women appeared to have opted for MRgFUS than the other two alternatives. More specifically, 71% of the patients aged between 45 and 54 opted for MRgFUS as opposed to 50% and 12% for UAE and myomectomy in this age group (Table 4). No woman below the age 34 opted for MRgFUS, which could be due to the fact that data regarding post-MRgFUS pregnancy is limited and provider counseling tends to recommend the most established option when childbearing is desired [27]. Furthermore, older age has been found to be associated with better success rate for MRgFUS, which might have factored in the decision regarding treatment options [28].

Although not modeled due to lack of data, the extent of potential loss in indirect income can be gauged from the fact that at least 64% of the women in the study worked with an employer contributing data to the MarketScan database. The remaining 36% of the women who opted to be in their spouses’ health plans might have also been employed elsewhere but the database did not capture their employment status. (Table 4) Since MRgFUS affords significantly faster recovery to normal activity [29-31], lost income due to missed workdays can be significantly lower for the MRgFUS cohort. Another notable finding was that significantly higher proportions of women undergoing MRgFUS lived in zip codes with the highest quintile of black residents. Because of the disproportionate impact of uterine fibroids on Black women, the acceptability of MRgFUS to this high-risk group may benefit from further investigation.

The secondary outcome in our study, UF-related cost for each treatment group, was estimated using matched comparison group without fibroids. Unlike the comparison groups for myomectomy and UAE, a more limited set of variables was used to select the comparison group for MRgFUS due to its small sample size. Specifically, a summary of comorbid conditions instead of the actual comorbid conditions was used in matching. This approach could potentially select a healthier comparison group for MRgFUS than for myomectomy and UAE. If so, the UF-related costs for MRgFUS may have been overestimated.

In the current environment where healthcare cost containment has become the guiding principle for any healthcare decision in the U.S., uptake of a new technology hinges not only on its superior clinical efficacy and safety profile but also on its cost-effectiveness compared to the existing alternatives. Clinical safety and effectiveness of MRgFUS with regard to symptom relief, fibroid shrinkage, adverse events and recurrence of symptoms and/or recourse to subsequent procedures have been shown in many studies worldwide [8,9,11,12,27,32-36]. However, its cost compared to relatively established alternative procedures for UF treatment, UAE and myomectomy, has not been widely documented. The only NIH-funded comparative effectiveness trial comparing clinical and economic outcomes associated with MRgFUS and UAE will not be available for several years [37]. One important reason for this dearth of comparative cost data has been due to the fact that the majority of the UF patient population is expected to be covered by commercial health insurance plans but most commercial insurance health plans in the U.S. consider MRgFUS as an experimental procedure [15-17]. Thus, MRgFUS is either not covered or it is paid for by some insurance plans only on a case by case basis. Consequently, it is difficult to gather reasonably large sample size of MRgFUS patients with cost data, which is particularly true for single-payer claims databases. Thus, the small sample size for MRgFUS cohort in our study is a reflection of the current reimbursement environment, and represents real-world evidence. The multi-payer database used in this study was perhaps one of the best data sources available at the time of initiating the study, which had 48 unique patients from across the U.S. who underwent MRgFUS. Other comparable commercial claims databases, including Comprehensive Health Analytics (A Humana Company) database and IMS LifeLink Database, had either no MRgFUS patients or had very small number of unique MRgFUS patients.

Standard limitations of an observational study apply to our study as well [38], which we sought to minimize through appropriate multivariate adjustments. As with any claims-based studies, our study modeled reimbursed amounts, which did not take into account different health plan designs, including deductible and copayment, which might affect patient’s choice of a specific procedure. Finally, since insurance claims do not record clinical variables including fibroid size and symptoms, analysis on the basis of these key variables was not possible.

## Conclusions

In summary, this study found that, after adjusting for patient characteristics, 1-year all-cause and UF-related costs were not different between patients undergoing MRgFUS, myomectomy and UAE. Restricting access to MRgFUS to constrain health care costs does not appear to be justified. More experience with MRgFUS can further refine the cost estimates that our study found.

### Condensation

Regression-adjusted one-year all-cause and uterine fibroid-related costs are not statistically different between patients undergoing magnetic resonance-guided focused ultrasound, myomectomy and uterine artery embolization.

## Competing interests

Dr. Stewart reports the following disclosures: InSightec Inc. (Research funding – paid to Mayo Clinic for patient care costs); Abbott, GlaxoSmithKline, Gynesonics, Bayer Health Care – Consulting. Other authors do not report any conflict of interests.

## Authors’ contributions

BB conceived of the study, contributed to study design, statistical analysis, interpretation of data, and drafted the manuscript. GC contributed in study design, data acquisition and interpretation of data. BM contributed to study design, data acquisition and interpretation of data. TG contributed to study design, data acquisition and interpretation of data. JM contributed to statistical analysis and interpretation of data. ES contributed to study design and interpretation of data. All authors read and approved the final manuscript.

## Meeting presentation

Preliminary results were presented at the Focused Ultrasound Foundation 3^rd^ International Symposium on Focused Ultrasound, October 14–17, 2012, Washington, DC Metro Area, USA.
